# Do Antibacterial Skin Sutures Reduce Surgical Site Infections After Elective Open Abdominal Surgery?—A Prospective, Randomized Controlled Single-Center Trial

**DOI:** 10.3390/jcm13226803

**Published:** 2024-11-12

**Authors:** Daniel Matz, Saskia Engelhardt, Andrea Wiencierz, Savas Deniz Soysal, Heidi Misteli, Philipp Kirchhoff, Oleg Heizmann

**Affiliations:** 1 Department of General, Visceral and Thoracic Surgery, Agaplesion Diakonieklinikum Rotenburg, 27356 Rotenburg (Wuemme), Germany; saskia.engelhardt@diako-online.de (S.E.); o.heizmann@diako-online.de (O.H.); 2 Clinical Trial Unit, University Hospital of Basel, 4031 Basel, Switzerland; andrea.wiencierz@usb.ch; 3 Faculty of Medicine, University of Basel, 4056 Basel, Switzerland; savas.soysal@hin.ch; 4 Clarunis, University Center for Gastrointestinal and Liver Diseases, 4056 Basel, Switzerland; heidi.misteli@spitaluster.ch; 5 Department of Surgery, Hospital Uster, 9610 Uster, Switzerland; 6 Zentrum für Hernienchirurgie und Proktologie, ZweiChirurgen, 4056 Basel, Switzerland; kirchhoff@zweichirurgen.ch

**Keywords:** surgical site infection (SSI), triclosan, suture, laparotomy, elective open surgery

## Abstract

**Background/Objectives**: The general use of triclosan-coated suture material (TCSM) for wound closure to prevent surgical site infections (SSIs) remains controversial. There is no conclusive evidence in the literature to support this and recommendations by professional organizations are contradictory. Therefore, the main objective of the study was to evaluate the 30-day rate of surgical site infections (SSIs) after elective open abdominal surgery using triclosan-coated versus uncoated running sutures (NCSM) for skin closure. **Methods**: This prospective, randomized, double-blinded study enrolled patients scheduled for open elective abdominal surgery, intraoperatively assigned to either the use of triclosan-coated or non-coated sutures for skin closure. The follow up was 30 days after surgery to detect SSIs. Secondary endpoints were wound dehiscence and reoperation rate due to wound dehiscence within 30 days, all-cause 30-day mortality and length of hospital stay. Potential risk factors for poor wound healing were evaluated in multivariate analysis. Data were analyzed in an intention to treat analysis. **Results**: In total, 364 patients (171 males [47%]) were randomized, 182 in either group. Six underwent urgent reoperation prior to first visit and were excluded from analysis. In the full analysis set (FAS), 358 were analyzed. SSI within 30 days occurred in 22 [12.2%] patients in the control group compared to 32 [18%] in the study group. The risk difference was not statistically significant (5.8%; 95% confidence interval (CI) −1.6–13.2%; *p* = 0.128). The wound dehiscence rate within 30 days was 14 of 179 [7.8%] in the NCSM group vs. 19 of 178 [10.7%] in the TCSM group. The difference in re-operation rates due to wound dehiscence was 0 of 179 [2.8%] vs. 5 of 178 [2.8%] in either group and not statistically significant (*p* = 0.0706). Among all patients recruited, 8 died within 30 days after surgery. Three of them died before the first assessment of the primary endpoint on day 3 and were therefore excluded from the FAS. The 30-day mortality rate was 2 of 180 [1.1%] in the NCSM group vs. 3 of 178 [1.7%] in the TCSM group. The majority of SSIs occurred in the superficial layer of the wound in both study groups (8.9% vs. 9.6%). The median [inter quartile range (IQR)] length of hospital stay was 13 [9.0, 19.2] days in the NCSM group vs. 11 [9.0, 16.8] days in the TCSM group There was a tendency towards shorter hospitalization in the study group (0.72 days [6%]). **Conclusions**: Our prospective randomized controlled trial could not confirm the superiority of TCSM for skin closure after elective open abdominal surgery in terms of SSI rates in a 30-day follow up period. Therefore, based on our results, a general recommendation for its use in all surgical fields cannot be justified.

## 1. Introduction

SSIs remain one of the most common complications following surgery, with an incidence of up to 19–20% [1,2] in a hospital setting. Numerous factors [3] have been found to influence postoperative wound healing, whereby patient-related factors such as gender, age, body mass index (BMI), smoking, pre-existing diseases (e.g., diabetes or arterial hypertension) vs. non-patient-related factors such as perioperative antibiotic prophylaxis and its timing, duration of operation, intraoperative blood loss, surgical technique, open vs. minimally invasive surgery, type of surgery (e.g., plastic, gynecological, colorectal or vascular surgery) and applied suture material can be distinguished. It can be assumed that these factors interact or even potentiate themselves [4].

It has been reported that percutaneous sutures approximating skin edges were often colonized from the body surface into the wound track by strains of Staphylococcus epidermidis capable of producing an amorphous extracellular matrix (biofilm), protecting the microbial populations from host defense factors [5]. Triclosan [2,4,4-trichloro-2-hydroxydiphenyl ether] is an antimicrobial agent that has been shown to reduce bacterial load in a wound and slow bacterial growth by inhibiting fatty acid synthesis. It is claimed that the coating provides protection against bacterial colonization of the tissue around the suture for almost 30 days and prevents the formation of a ligature abscess. In vitro studies have shown that triclosan forms a zone of inhibition around the suture material and is effective against the most common pathogens of SSIs, particularly Gram-positive bacteria [6].

The use of triclosan-coated suture material in surgical health care is widespread and considered safe. This might be a reflection of different surgical guidelines or guidance advocating its use in the intention to reduce postoperative SSIs.

In 2016, the WHO [7] published their recommendations on intraoperative and postoperative measures for surgical site infection prevention, endorsing a collection of measures to achieve the goal of lower SSI rates, as did the Centers of Disease Control (CDC) in 2017 [8]. In both publications, the use of TCSM is advocated. The 2017 CDC guideline recommends considering this as a weak recommendation, suggesting a trade-off between clinical benefits and harms, whereas the authors of the WHO recommendation advocate routine use of TCSM independently of the type of surgery as a conditional recommendation. Importantly, both recommendations were based on a moderate level of evidence. The latter is mainly based on the meta-analysis by Wu et al. [9]. Comparable with recently published meta-analyses addressing the impact of triclosan-coated suture material in wound closure for the prevention of SSIs, different types of surgery (breast, vascular, orthopedic, colorectal [10,11,12,13]) were analyzed, and emergency surgery was compared to elective surgery and open surgery was compared to laparoscopic surgery in the publications mentioned afore. Remarkably, the use of TCSM is recommended when available for wound closure in clean and clean-contaminated abdominal cases according to The American College of Surgeons (ACS)/ Surgical Infection Society (SIS) Guidelines 2016 Update [14]. These inconsistent recommendations reflect the lack of profound evidence in this field, although numerous studies have attempted to clarify the plausibility of using TCSM in different types of surgery. Thus, The Society for Healthcare Epidemiology of America and the Infectious Diseases Society of America practice do not recommend the routine use of TCS [1].

In contrast, in 2017, the U.S. Food and Drug Administration (FDA) issued a final recommendation [15] determining that triclosan and 23 other active ingredients are not generally recognized as safe and effective for use in certain over-the-counter health care antiseptic products because no additional safety and effectiveness data for these ingredients were provided to the agency.

Therefore, the aim of our prospective randomized, double-blinded single-center study was to compare postoperative SSI rates in a tertiary care teaching hospital for patients undergoing elective open abdominal surgical procedures from February 2016 to October 2020 using triclosan-coated or uncoated suture materials for skin closure. The study protocol was published in 2019 in the *Trials* journal [16].

## 2. Materials and Methods

Between February 2016 and October 2020, this single-center prospective randomized controlled trial was conducted at the tertiary teaching hospital Agaplesion Diakonieklinikum Rotenburg, Rotenburg (Wuemme), Germany, in the Department of Visceral and Thoracic Surgery and the Department of Obstetrics and Gynecology, designed as a double-blinded, parallel-group superiority trial. The study protocol followed the Standard Protocol Items: Recommendations for Interventional Trials (SPIRIT) guidelines [17]. The trial was approved by the Ethics committee of the University of Goettingen, Goettingen, Germany. Trial registration: germanctr.de, Identifier: DRKS00010047.

### 2.1. Patients

Inclusion criteria: Patients scheduled for open elective, either general/colorectal or gynecological, surgery for malignant or non-malignant disease aged 18 years or older were subsequently enrolled in the trial after written consent. Open abdominal surgery is defined as a surgically opened peritoneal cavity.

The following criteria were defined es exclusion criteria:

- Participation in a clinical study in the last 3 months;
- Factors limiting the ability to co-operate in the study;
- Absence of signed informed consent before entering the study;
- Any drug, alcohol, or nicotine abuse;
- People with mental disorders;
- Pregnant women;
- Participants under 18 years;
- Emergency surgery/procedures.

Baseline characteristics such as age, sex, BMI (body mass index), presence of diabetes mellitus, immunosuppression, ASA (American Society of Anesthesiologists) classification, length of incision, orientation of incision, CDC wound classification, duration of operation, blood loss during operation, type of procedure (colorectal procedure: yes/no; resection of tumor: yes/no) and department performing the operation (gynecology/general surgery) were recorded.

### 2.2. Definition and Diagnosis of SSIs

SSIs are defined as either superficial, deep or organ-spaced infection according to CDC criteria [18]. Superficial incisional SSIs include skin and subcutaneous tissues, deep incisional infections involve fascia and muscle, and organ-spaced infections involve any organ or space other than the incised layer of body wall that was opened or manipulated during surgery. The following criteria for the diagnosis of an SSI were used:

The infection occurs within 30 days after the primary operation or within 1 year if an implant is in place and the infection appears to be related to the operation and involves skin or subcutaneous tissue, deep soft tissues (e.g., fascial and muscle layers) or any part of the anatomy (e.g., organs or spaces) and at least one of the following: (1) purulent drainage from the wound, organisms detected by wound swab, diagnosis clinically or at imaging, or wound opened spontaneously, or by a surgeon or attending physician and (2) the patient had at least one of the following: pain, tenderness, localized swelling, redness, heat at the wound site, or systemic fever (>38 °C).

### 2.3. Randomization and Intervention

Patients were assigned intraoperatively to either the study group (triclosan-coated suture material (TCSM) group) or the standard protocol group (non-coated suture material (NCSM) group).

The randomization sequence was performed using opaque sealed envelopes containing the suture material for skin closure. Randomization of the study groups was performed block wise (allocation 1:1) using block sizes of 20 items per block. The randomization list was generated by a computer using the Random Allocations Software Version 1.0.0 (Freeware) by M. Saghaei, MD.

In the TCSM group, triclosan-coated poliglecaprone 25 (Monocryl plus, Ethicon GmbH, Norderstedt, Germany) was used for skin closure; in the NCSM group, uncoated poliglecaprone 25 was used (Monocryl).

### 2.4. Surgical Procedure

After administration of perioperative antibiotic prophylaxis (standard dose of first- or second-class cephalaosporine 30–60 min prior to surgery), wound opening was performed in layers according to the following steps:

- Skin incision with scalpel;
- Subcutaneous preparation with monopolar cautery, scalpel or scissors;
- Incision of muscular fascia with monopolar cautery, scalpel or scissors.

When the surgical procedure itself was completed, skin closure was performed in layers with running sutures for fascia with an Everett suture (Maxon 1, Covidien IIC, Mansfield, MA, USA). No subcutaneous sutures or drains were applied.

Immediately before wound closure, the randomization envelope was opened by an unscrubbed nurse, not involved in the follow up of the patient. The same nurse subsequently gave the suture material to the scrub nurse. The sutures were handed over to the surgeon without showing the package of the suture material. Therefore, the patient and the surgeon were unaware of the suture material used, as it is impossible to distinguish between the two suture types macroscopically. After closing the skin incision with running sutures, a sterile wound dressing was applied.

### 2.5. Follow up

All patients were followed up for 30 days after surgery according to the CDC guidelines. Routine wound surveillance was performed including diagnosis and treatment of SSIs. On day 3 and 7, wound photographs were taken in terms of surveillance by the resident in charge who was also blinded to the used surgical suture. When an SSI was detected by a resident, it had to be verified by the consultant surgeon in charge. Furthermore, inpatients were routinely seen by the study team as well. The 30-day follow up visit, including photographic wound documentation, was performed by a well-trained (blinded) investigator. In general, when an SSI was detected during study follow up, wound swabs were taken and standard wound therapy was applied or a re-operation performed, as necessary. A one-year follow up was performed in case of an implanted foreign body. To provide appropriate specificity, all detected cases of SSIs were validated by a board-certified wound care specialist.

### 2.6. Outcomes

The primary outcome measure was the occurrence of any SSI during the 30-day follow up period after primary surgery or after one year of follow up when a foreign body was implanted. Only primary incisions at the time of the index operation were evaluated for SSIs.

The secondary outcome measures were wound dehiscence and re-operation rate due to wound dehiscence within 30 days, all-cause 30-day mortality, length of hospital stay and the occurrence of SSI one year after surgery when a foreign body was implanted.

Additionally, potential baseline characteristics for poor wound healing such as gender, age, BMI, ASA classification, diabetes mellitus, present immunosuppression, wound class according to CDC criteria, operative time, amount of blood loss, length and orientation of incision, type of procedure (colorectal vs. none colorectal), department performing operation (gynecology vs. general surgery) and tumor resection vs. non tumor resection were evaluated.

## 3. Statistical Methods

### 3.1. Sample Size Calculation

The sample size was estimated on the basis of a two-sided chi^2^ test. Based on evidence in the literature, an infection rate of 12% was assumed for the control group and a reduction in the SSI rate to 4% in the treatment group. Refs. [2,19] Given a test level of α = 0.05, a total of 344 patients would be needed to show that the SSI rates are different with a power of at least 80%. Accounting for an expected drop-out rate of 5%, *n* = 364 study participants were recruited, with 182 in either study group.

### 3.2. Statistical Analysis

Statistical analysis was performed from January to September 2021. All analyses were performed in R version 4.1.1 (2021-08-10) or higher. For the primary analysis, a two-sided chi^2^ test was performed at a significance level of 5%. In addition to the primary analysis, differences in SSI rates in the treatment groups were estimated using a multiple logistic regression model, controlling for the following general risk factors for poor wound healing: age, BMI, length of incision, orientation of incision, CDC wound classification, duration of operation, amount of blood loss, and colorectal procedure (yes/no).

Furthermore, differences regarding the following secondary endpoints were assessed: wound dehiscence within 30 days, re-operation due to wound dehiscence within 30 days, death within 30 days, SSI classification according to CDC criteria, and length of hospital stay.

The first three secondary endpoints are binary outcomes and were therefore analyzed estimating simple and multiple logistic regression models with explanatory factor treatment and, in the multiple case, adjusted for the before mentioned general risk factors for poor wound healing.

The secondary endpoint SSI classification is an ordinal outcome and was analyzed using an ordered logistic regression model. Instead, the endpoint length of hospital stay (measured in days) is a positive discrete variable and was investigated using negative binomial regression models. The analyses of length of hospital stay were restricted to the subset of patients discharged from hospital alive.

All statistical analyses were performed on the FAS according to the intention to treat principle. The FAS contained all study participants who were randomized and for whom data of at least one follow up visit were available. There were 12 patients in the FAS who had no SSI on day 3 after surgery and for whom the assessments of SSI on day 7 and/or day 30 were missing. For these patients, it is not known if they had an SSI within 30 days. Therefore, primary endpoint and the closely related secondary endpoint SSI classification suitable imputation models were used. More detailed explanations are given in Appendix A.

All data were collected in an anonymous and encrypted database by the investigator at the Agaplesion Diakonieklinikum Rotenburg (Wuemme), Germany.

## 4. Results

**Figure 1:**
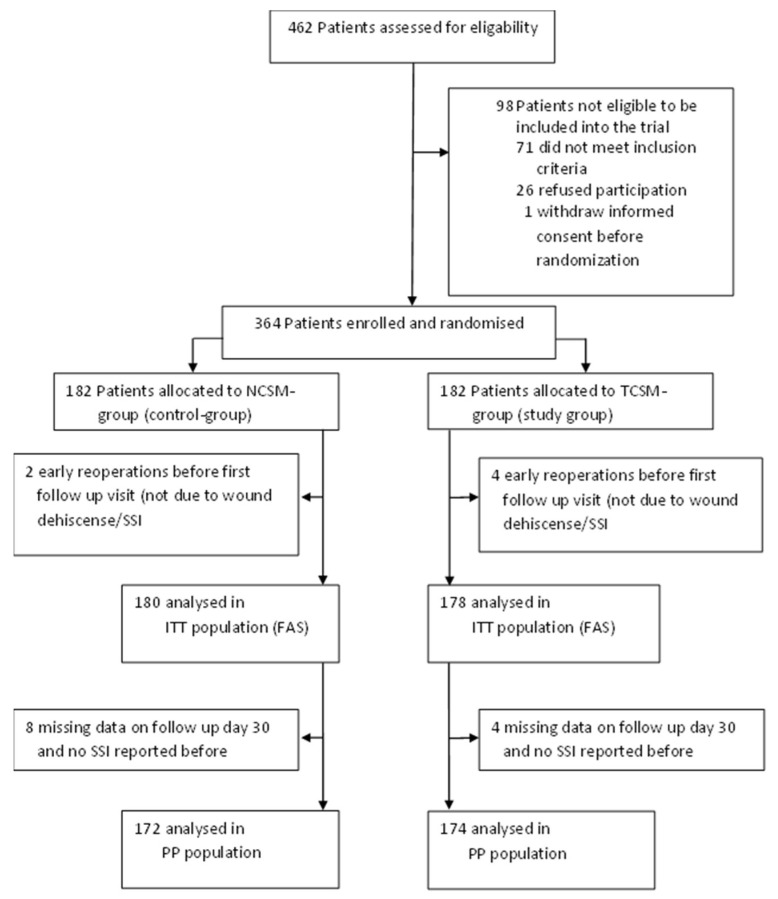
CONSORT flow diagram: patient flow according to Consolidated Standards of Reporting Trials (CONSORT) [20] including all reasons for exclusions from the intention to treat (ITT) and the per protocol (PP) population. NCSM, non-coated suture material; TCSM, triclosan-coated suture material; SSI, surgical site infection; FAS, full analysis set; PP = per protocol.

In total, 364 patients were enrolled in the study and randomized to one of the two treatment groups. The CONSORT flow diagram is shown in Figure 1. Of the 364 randomized patients, 6 patients had no data on SSIs at any of the three follow up visits due to re-operation within the first 3 days after randomization. Thus, the FAS contains data of 358 patients, where 180 were randomized in the standard group and 178 in the study group.

### 4.1. Patient Characteristics

The baseline characteristics of the study groups are shown in Table 1; both are well-balanced regarding their baseline characteristics. Overall, 169 patients (47.2%) were males and 189 (52.8%) were females, with a mean age of 67.7 (standard deviation (SD): 12.8 years). The median BMI was 25.5 [inter-quartile range (IQR): 23.1, 29.0] kg/m^2^, corresponding to overweight according to the WHO classification. In total, 67 (18.7%) participants were diabetic and 12 (3.4%) were immunosuppressed. The majority of study participants had a severe systemic disease according to the ASA classification (ASA stage I, 13 (3.6%); ASA stage II, 166 (46.4%); ASA stage III, 179 (50.0%)).

### 4.2. Operative Details

The mean incision length (SD) was 24.7 (6.8) cm, and the most performed laparotomy was a transverse (midline) incision, on 191 patients (53.4%). In more than two thirds of patients, a clean-contaminated situs was present, for 243 patients (67.9%), according to CDC classification. The median intraoperative blood loss (median [IQR] 200.0 [100.0, 500.0] mL vs. median [IQR] 200.0 [100.0, 400.0] mL) and duration of operation (median [IQR] 195.5 [145.8, 296.2] min vs. median [IQR] 197.5 [145, 285] min) were comparable in either group. Overall, 148 patients (41.3%) were colorectal cases, and in 305 (85.2%) patients, a tumor was resected. The minority of cases were gynecological procedures (*n* = 48 (13.4%), with no cesareans included.

### 4.3. Primary Analysis

The endpoint data of all patients in the FAS classified by treatment group are given in Table 2. After using model-based imputation for 12 patients with missing endpoint data in the FAS, the 30-day SSI rate was higher in the TCSM group (18% [95% CI, 13–24.3]) vs. the NCSM group (12.2% [95% CI, 13–24.3]), (*p* = 0.128).

The sensitivity analyses considering alternative imputation methods led to the same conclusions. The corresponding tables can be found in the Supplementary Materials (Tables S1–S3).

In the secondary analysis, differences in SSI risk between the treatment groups were assessed using univariate and multiple logistic regression, with the latter adjusting for potential confounders of poor wound healing. The estimated odds ratios (ORs) and 95% CIs from multivariable logistic regression are displayed in Table 3. The unadjusted model has a lower Akaike’s information criterion (AIC, 305.4 vs. 307.3) and thus fits better to the data, reflecting well-balanced treatment groups with respect to potential confounders. The OR (1.57 [95% CI, 0.87–2.83]) estimated in the unadjusted model suggests a 57% higher risk of experiencing an SSI within 30 days for patients in the TCSM group. However, the corresponding 95% CI is rather wide, reflecting a higher degree of statistical uncertainty. Therefore, the data are not conclusive about which treatment involves a higher risk for SSI within 30 days. Sensitivity analyses considering alternative imputation methods led to the same conclusions. The corresponding tables can be found in the Supplementary Materials (Tables S4–S9).

Table 4 shows the results of the secondary endpoints assessed for confounding factors of poor wound healing using univariate and multiple logistic regression models with model-based imputation.

One patient was lost to follow up and therefore had missing information regarding secondary endpoints including wound dehiscence, re-operation due to wound dehiscence and death within 30 days. As such, the analysis was based on 357 patients for whom all data were available for these three endpoints.

The wound dehiscence rate within 30 days was 14 of 179 [7.8%] in the NCSM group vs. 19 of 178 [10.7%] in the TCSM group (Table 2). After controlling for potential confounding due to factors for poor wound healing, there was no clear evidence of a difference between the groups in the adjusted model (OR 1.19 [95% CI, 0.56, 2.53]), while in the univariate model, there seemed to be a slight tendency in favor of the NCSM group (OR, 1.41 [95% CI, 0.68–2.90]). These results do not change when applying best-case or worst-case imputation to the missing data of one patient.

Five patients underwent a re-operation, all of them in the TCSM group. Therefore, the regression models could not be reliably estimated. The difference in re-operation rates due to wound dehiscence was not statistically significant in a chi^2^ test with continuity correction (*p* = 0.0706), based on all 357 patients with available endpoint data. Assuming that the patient lost to follow up underwent a re-operation, the logistic regression models could be estimated, confirming the results of the chi^2^ test.

Consistently, chi^2^ tests (with continuity correction) based on the worst-case and best-case imputations of the endpoint data of the one patient lost to follow up do not point out a statistically significant difference between the groups (*p* = 0.212; *p* = 0.0697).

Among all recruited patients, eight died within 30 days after surgery. Three of them died before the first assessment of the primary endpoint on day 3 and therefore were excluded from the FAS. According to the sensitivity analysis, these exclusions were not critical. In total, 2 of 180 [1.1%] patients in the NCSM group and 3 of 178 [1.7%] patients in the TCSM group died within 30 days after operation. The OR in the univariate analysis (unadjusted model) was 1.52 [95% CI, 0.25–9.19] and the CI was rather wide, including values below 1. The unadjusted model fits better to the data (AIC [56.4 vs. 58.7]). Best- and worst-case imputation of the one missing value yielded results in line with those above.

The reasons for postoperative mortality were immediate postoperative multiorgan failure (in four patients), myocardial infarction, pneumonia, and hemorrhagic shock due to severe bleeding. Bilateral pulmonary embolism occurred in the remaining case. All aforementioned patients were suffering from malignant tumors and therefore underwent pancreatic head resection in three cases and esophageal, gastric, hepatic, colonic, and abdominal sarcoma resection one patient each.

In no cases was there a relationship observed regarding the skin suture material used in the trial.

Most frequently, SSIs were detected superficially in either group (16 [8.9%] NCSM group vs. 17 [9.6%] TCSM group). No deep SSIs occurred in the NSCM group vs. 3 [1.7%] in the TCSM group. The number of organ-spaced SSIs was lower in the NCSM group, at 6 [3.3%] vs. 10 [5.6%]. The OR in the univariate analysis with model-based imputation of the SSI classification (*n* = 358) was 1.6 [95%CI, 0.89–2.87]. The fully adjusted model could not be reliably estimated. Therefore, it was adjusted only for the four major risk factors of poor wound healing: age, BMI, length of incision and orientation of incision. The unadjusted model fitted better to the data AIC [395.8 vs. 398.1]. Sensitivity analyses considering alternative imputation methods led to the same conclusions. The corresponding tables can be found in the Supplementary Materials (Tables S10–S13).

Evaluating the length of hospital stay, all those patients in the FAS were included, for whom it is known that they were discharged from hospital alive (*n* = 352). There was a weak tendency for longer hospital stay in the NCSM group, with a 6% (OR 0.94 [95%CI, 0.84–1.04]) shorter length of hospital stay (=0.72 days) on average according to the adjusted model. Moreover, the results of this secondary analysis show that the length of hospital stay indeed depends on general patient characteristics and risk factors, since the adjusted model clearly yields a better model fit according to the corresponding AIC [adjusted model 2364.6 vs. AIC unadjusted 2499.5]. 

Apart from this secondary endpoint, the remaining data appeared to not be confounded by potential risk factors of poor wound healing.

Moreover, we analyzed whether the treatment effect on SSI varies between tumor patients and patients who underwent a different surgery. Using a likelihood ratio test at the 5% level, we compared a logistic regression model with SSI as the outcome and the treatment arm as the sole predictor with a model including an additional interaction term with a variable indicating whether a tumor resection was performed. The subgroup analysis did not find evidence that the treatment effect on SSI occurrence varies between tumor patients and patients who underwent a different surgery. The *p*-value of the corresponding likelihood ratio test was 0.827 (using the FAS with model-based imputation of the primary endpoint).

In a post hoc subgroup analysis, we investigated whether the treatment effect varies between men and women. Indeed, adding an interaction term between arm and sex to the multivariable model shown in Table 3 yielded a lower AIC (304.4) than the other model in Table 3 (307.3). This suggests that, even when accounting for general risk factors of poor wound healing, men and women may respond differently to the sutures used.

We repeated all analyses using the PP population to assess the robustness of the above findings. The results of the primary analysis are displayed in the Supplementary Materials (Tables S14 and S15) and are perfectly in line with the FAS analyses.

Finally, none of the nine patients in whom a foreign body was implanted during the surgical intervention developed an SSI in the one-year follow up period.

## 5. Discussion

Contrary to what had been expected, the SSI rate was higher in patients receiving antibacterial-coated sutures than in the control group (18% vs. 12.2%). However, this difference was not statistically significant. Considering the secondary outcomes, we found a slight trend towards a higher risk of experiencing an SSI within 30 days after surgery among patients in the antibacterial suture arm than in the control arm. Regarding the length of hospital stay, the results suggest a small benefit of antibacterial sutures, with minimally shorter lengths of hospital stay according to the analysis model that was adjusted for confounding.

Olmez et al. [21] found a reduction in SSIs using triclosan-coated polydioxanone (PDS) for fascial closure that was as high as 24% in comparison to uncoated PDS. Elective cases and patients requiring urgent gastrointestinal surgery were included in that study. The rate of SSI in the study group (19.1%) is comparable to our results, but the SSI rate of 25.8% in the standard group is surprisingly high compared to the 12.2% rate of our study and previous publications [1,2].

In a more recent double-blinded RCT conducted by Kang et al. [22], no reduction in surgical site infections within 30 days after colorectal surgery (open and laparoscopic) could be demonstrated. Interestingly, the rate of SSIs after 30 days differed significantly (7.1% in the triclosan-coated group vs. 28.0% in the control group). It seems unlikely that the reduction in SSIs observed after 30 days was attributable to TCSM, given the pharmacodynamic findings of Daoud et al. [23]. In vitro time-kill assays were conducted with eight triclosan-susceptible microorganisms common in surgical site infections. The results demonstrated a consistent pattern, with an initial rapid decline in microbial concentration from the baseline to 4–8 h, followed by a plateau up to 24 h in the majority of cases.

Given that the proportion of colorectal procedures in our study was 40.9% (NCSM group 41.8% vs. TCSM group 40.1%) and that colorectal surgery has been identified as a potential risk factor for postoperative SSIs in recent studies, corresponding meta-analyses are of interest. Hunger and colleagues [3] demonstrated a reduction in SSIs in monocentric trials only, whereas a positive effect could not be confirmed in multicentric trials (OR = 1.75, 95% CI [1.11–2.77]). In the evidence review for the effectiveness of wound closure materials and techniques in the prevention of surgical site infection of the NICE guideline NG125 [24], a similar result was found focusing on colorectal procedures (test for overall effect: Z = 1.29 (*p* = 0.35)). Weiser and colleagues [25] impressively demonstrated a reduction in postoperative SSIs in colorectal surgery due to the implementation of a multidisciplinary bundle of care in the Memorial Sloan Kettering Cancer Center, New York, USA, from 11% to 4.1% without the usage of TCS for wound closure. These and the results of our secondary analysis controlling for general risk factors for poor wound healing including colorectal procedures indicate that TCSM is unlikely to exert a significant positive influence on the SSI rate for colorectal procedures.

The only multicountry randomized clinical study conducted (FALCON trial [26]) revealed no benefit from 2% alcoholic chlorhexidine skin preparation compared with povidone–iodine, or with triclosan-coated sutures compared with non-coated sutures, in preventing SSIs in clean-contaminated or contaminated or dirty surgical wounds. However, this study was conducted in low- and middle-income countries, which means that the results cannot be easily extrapolated to industrialized countries with more efficient health care systems, but this result clearly contradicts the WHO’s general recommendation to use TCSM for wound closure in all types of surgery. The generalizability of this recommendation is also debatable with regard to the meta-analyses by Elsohl and colleagues [27] evaluating the effect of antibiotic-impregnated sutures for abdominal wall closure, as they could not provide further evidence to support the effectiveness of TCSM in terms of SSI reduction. The risk of SSIs in the antibiotic-impregnated suture group (all sutures in the selected studies were TCS) was 10.4% vs. 13.0% in the control group (odds ratio of 0.79, 95% CI [0.57–1.09], *p* = 0.15). The SSI-reducing effect of TCS found in the meta-analysis by de Jonge [11] could not be verified in the subgroup of high-quality studies.

The 2021 NICE guidance [28], as a result of a commissioned external assessment center (EAC) [29] of submitted evidence by the manufacturer of TCS, recommends the use of TCS as a part of a strategy preventing SSIs. This is based on 31 RCTs comparing the use of triclosan-coated sutures with their non-coated counterparts. The most represented category in these trials was gastrointestinal or abdominal surgery (nine RCTs [21,30,31,32,33,34,35,36,37]). None of the three trials [31,32,35] categorized as high-quality studies (consistent with the GRADE methodology) could demonstrate the effectiveness of reducing postoperative SSIs, which aligns with the meta-analysis provided by the EAC, when only high-quality studies were included in the analysis. Finally, the positive effect of the above meta-analyses could not be confirmed in the subgroup analyses or by considering only high-quality trials.

Based on results from recent cohort studies, where the majority of postoperative SSIs were localized superficially [38,39], and the fact that organ-spaced infections usually result from anastomotic dehiscence or leak [25] and are thus minimally influenced by the suture material used for abdominal wall closure, we aimed to apply the antibiotic-impregnated suture material at the level of the skin in our trial. This also contrasts with all studies included in one of the most recent meta-analyses [40] where the TCSM was applied at the fascial level. In both of the study groups we evaluated, most SSIs were found in the superficial layer (NCSM group 8.9% vs. TCSM group 9.6%). However, after adjustment for general risk factors for poor wound healing in the secondary analysis, no confounding of the SSI classification due to these risk factors could be demonstrated. In comparison, in the PROUD Trial by Diener et al. [31], the coated suture material was used in the fascial layer. They found an overall SSI rate of 14.8% in the study group vs. 16.1% in the control group, but the difference was not statistically significant (*p* = 0.64). In line with our results, most of the SSIs appeared superficially (9.0% vs. 9.4%). Mattavelli et al. [35] used triclosan-coated sutures in all layers during closure of the laparotomy (peritoneum, fascia and skin). The prevalence of superficial SSIs was 4.9% (7/141) in the standard group vs. 10% (14/140) in the study group (*p* = 0.115). They found no statistical difference in the occurrence of SSIs between the groups. Remarkably, Lin et al. [41] analyzed the influence of TCS on inflammatory markers and wound surface temperature in patients undergoing total knee arthroplasty in a prospective double-blind randomized controlled trial. The suture material was utilized in all layers of wound closure (arthrotomy, fascial layer and subcutaneous) apart from the skin edges, which were stapled. Except for IL-6, the inflammatory markers were not significantly different between the triclosan and control groups. However, the wound temperature was significantly lower 3 months post operation in the study group. Importantly, these findings did not result in a statistical difference in the postoperative SSI rate of 3.9% in the control vs. 0% in the study group (*p* = 0.495%).

Considering these results, it seems that the experimental effects found in in vitro or animal tests [23,42,43] showing significant inhibition of bacterial growth around the suture material due to triclosan impregnation cannot necessarily be reproduced or translated into in vivo results, since the rate of postoperative SSI was not influenced by the use of TCSM, independently of whatever anatomical layer the TCSM was used in, even when using it for all layers in wound closure.

Looking at the financial burden of postoperative SSIs, the shortened length of hospital stay we found as a tendency might be of potential benefit when using TCSM for skin closure after laparotomy. With a 6% (0.72 days), on average, shorter hospital stay in the adjusted model, this might be translated into a reduction in costs. Nevertheless, the results in the secondary analysis showed that the length of hospital stay mostly depends on general patient characteristics and risk factors, which has been proven in different studies [3]. Focusing on the length of hospital stay, it is likely that the additional expenses for the triclosan-coated suture material in open abdominal surgery are not offset by a measurable clinical benefit and might carry substantial cost implications in resource-limited settings, as mentioned by the authors of the FALCON trial.

## 6. Conclusions

In our prospective randomized double-blinded RCT for patients undergoing elective open abdominal surgery, we could not verify the suggested advantages of triclosan-coated sutures compared with uncoated sutures for skin closure regarding postoperative SSI rates. Therefore, based on our results, a general recommendation for its use in all surgical fields cannot be justified. The additional expenses for the coated suture material seem to not be offset by a measurable clinical benefit.

To further elucidate the influence of TCS on postoperative SSIs, multicentric prospective RCTs are needed, focusing on multidisciplinary bundles of care including the presence or absence of triclosan-coated sutures for wound closure, stratifying for type of surgery (such as colorectal procedures) and predicted patient-related risk for postoperative SSI. Furthermore, it would be advisable to focus on particularly vulnerable subpopulations such as immunosuppressed patients.

## 7. Limitations

Potential limitations could be due the unexpected number of SSIs found in the study group. The assumed SSI rate of 12% in the sample size calculation is in line with our result of 12.2%, whereas it is much higher in the treatment arm (18%) but is still in the range of other publications [1,2]. Furthermore, this study may also have limitations due to its single-center design. 

## Figures and Tables

**Table 1:** Baseline characteristics of patients in the FAS by treatment arm with standardized mean differences (SMDs).

	Standard Suture (*n* = 180)	Antibacterial Suture (*n* = 178)	SMD
Age (decimal years) (mean (SD))	67.0 (12.4)	68.5 (13.1)	0.119
Sex = male (%)	76 (42.2)	93 (52.2)	0.202
BMI (kg/m^2^) (median [IQR])	25.2 [22.5, 28.3]	25.9 [23.6, 29.4]	0.234
Diabetes mellitus = yes (%)	35 (19.4)	32 (18.0)	0.038
Present immunosuppression = yes (%)	6 (3.3)	6 (3.4)	0.002
ASA physical status classification (%)			0.049
1	7 (3.9)	6 (3.4)	
2	85 (47.2)	81 (45.5)	
3	88 (48.9)	91 (51.1)	
Length of incision (cm) (mean (SD))	24.6 (6.8)	24.9 (6.9)	0.045
Orientation of incision (%)			0.202
Transverse	105 (58.3)	86 (48.3)	
Longitudinal	61 (33.9)	74 (41.6)	
Both	14 (7.8)	18 (10.1)	
CDC wound classification (%)			0.085
Clean	50 (27.8)	56 (31.5)	
Clean-contaminated	125 (69.4)	118 (66.3)	
Contaminated/ dirty	5 (2.8)	4 (2.2)	
Duration of operation (min) (median [IQR])	195.0 [145.8, 296.2]	197.5 [145.0, 285.0]	0.027
Blood loss during operation (mL) (median [IQR])	200.0 [100.0, 500.0]	200.0 [100.0, 400.0]	0.067
Colorectal procedure = yes (%)	76 (42.2)	72 (40.4)	0.036
Department performing operation = gynecology (%)	22 (12.2)	26 (14.6)	0.070
Resection of tumor performed = yes (%)	149 (82.8)	156 (87.6)	0.137

Abbreviations: ASA, American Society of Anesthesiologists; BMI, body mass index (calculated as weight in kilograms divided by height in meters squared); CDC, Centers of Disease Control; IQR, interquartile range; SD, standard deviation.

**Table 2:** Endpoint data of all patients in the FAS by treatment arm before imputation.

	Standard Suture (*n* = 180)	Antibacterial Suture (*n* = 178)
SSI within 30 days (%) no	150 (83.3)	144 (80.9)
yes	22 (12.2)	30 (16.9)
NA	8 (4.4)	4 (2.2)
Wound dehiscence within 30 days (%) no	165 (91.7)	159 (89.3)
yes	14 (7.8)	19 (10.7)
NA	1 (0.6)	0 (0.0)
Re-operation due to wound dehiscence 30 days (%) no	179 (99.4)	173 (97.2)
yes	0 (0.0)	5 (2.8)
NA	1 (0.6)	0 (0.0)
Died within 30 days (%) no	177 (98.3)	175 (98.3)
yes	2 (1.1)	3 (1.7)
NA	1 (0.6)	0 (0.0)
SSI classification (%) no_SSI	150 (83.3)	144 (80.9)
superficial	16 (8.9)	17 (9.6)
deep	0 (0.0)	3 (1.7)
organ-spaced	6 (3.3)	10 (5.6)
NA	8 (4.4)	4 (2.2)
Length of hospital stay (days) (median [IQR])	13.0 [9.0, 19.2]	11.0 [9.0, 16.8]

Abbreviations: SSI, surgical site infection; Control; IQR, interquartile range.

**Table 3:** ORs and 95% CIs from multivariable logistic regression model based on FAS data with model-based imputation of the primary endpoint (*n* = 358).

	Odds Ratio [Yes vs. No]	95% CI
Antibacterial suture	1.47	[0.80, 2.70]
Age	1.03	[1.00, 1.06]
BMI	1.00	[0.94, 1.06]
Length of incision	1.03	[0.97, 1.09]
Orientation of incision—longitudinal	1.81	[0.90, 3.62]
Orientation of incision—both	1.72	[0.58, 5.12]
CDC wound classification—clean-contaminated	1.65	[0.69, 3.96]
CDC wound classification—contaminated/ dirty	0.00	[0.00, Inf]
Duration of operation	1.00	[1.00, 1.01]
Blood loss	1.00	[1.00, 1.00]
Colorectal procedure—yes	1.31	[0.60, 2.85]

Abbreviations: BMI, body mass index (calculated as weight in kilograms divided by height in meters squared).

**Table 4:** Results of univariate and multivariate regression analysis on the secondary outcome parameters.

	Secondary Endpoint—Univariate Analysis (Unadjusted Model)									
	Wound Dehiscence		Re-Operation Due to Wound Dehiscence ^a^		Death		SSI Classification		Length of Hospital Stay ^b^	
	OR	95% CI	OR	95% CI	OR	95% CI	OR	95% CI	Estimate	95% CI
	1.41	[0.68, 2.90]	5.17	[0.60, 44.73]	1.52	[0.25, 9.19]	1.6	[0.89, 2.87]	0.92	[0.82, 1.04]
	**Secondary Endpoint—Multivariate Analysis (Adjusted Model)**									
Antibacterial suture	1.19	[0.56, 2.53]	3.74	[0.40, 34.88]	2.42	[0.40, 34.88]	1.55	[0.85, 2.82]	0.94	[0.84, 1.04]
Age	1.05	[1.01, 1.09]	1.00	[0.93, 1.08]	1.14	[0.93, 1.08]	1.03	[1.00, 1.05]	1.01	[1.01, 1.01]
BMI	1.06	[0.99, 1.14]	1.05	[0.90, 1.21]	0.51	[0.90, 1.21]	0.99	[0.93, 1.05]	0.99	[0.98, 1.00]
Length of incision	1.03	[0.96, 1.11]	1.09	[0.91, 1.31]	0.94	[0.91, 1.31]	1.04	[0.99, 1.10]	1.01	[1.00, 1.02]
Orientation of incision										
- longitudinal	3.14	[1.30, 7.58]	3.90	[0.26, 57.99]	0.72	[0.26, 57.99]	1.37	[0.73, 2.58]	0.96	[0.85, 1.08]
- both	2.26	[0.55, 9.23]	2.85	[0.19, 43.04]	2.16	[0.19, 43.04]	1.34	[0.47, 3.83]	0.84	[0.69, 1.03]
CDC classification - clean-contaminated	1.74	[0.57, 5.30]	0.20	[0.02, 1.73]	0.12	[0.02, 1.73]		NA ^c^	1.19	[1.03, 1.37]
- contaminated/dirty	0.79	[0.07, 9.49]	0.00	[0.00, Inf]	0.00	[0.00, Inf]		NA ^c^	1.40	[0.98, 2.00]
Duration of operation	1.0	[1.00, 1.01]	1.01	[1.00, 1.02]	1.01	[1.00, 1.02]		NA ^c^	1.00	[1.00, 1.00]
Blood loss	1.0	[1.00, 1.00]	1.00	[1.00, 1.00]	1.00	[1.00, 1.00]		NA ^c^	1.00	[1.00, 1.00]
Colorectal procedure										
yes	1.24	[0.47, 3.29]	.95	[0.07, 12.54]	0.36	[0.07, 12.54]		NA ^c^	0.84	[0.73, 0.96]
AIC (unadj. vs. adj. model)	223.14 vs. 223.06		62 vs. 70.4		56.4 vs. 58.7		395.8 vs. 398.1		2499.5 vs. 2364.6	

Abbreviations: AIC, Akaike’s information criterion; BMI, body mass index (calculated as weight in kilograms divided by height in meters squared); CDC, Centers of Disease Control; CI, confidence interval; OR, odds ratio. A lower AIC indicates a better model fit for the corresponding model. ^(a)^ Worst case imputation model, assuming that the one patient who was lost to follow up had to undergo a re-operation. ^(b)^ Multiplicative effects and 95% CIs from negative binomial regression model based on FAS data of all those patients discharged from hospital alive (*n* = 352). ^(c)^ The fully adjusted model could not be reliably estimated. Therefore, adjustment only for the four major risk factors for poor wound healing age, BMI, length of incision and orientation of incision was estimated.

## Data Availability

Deidentified participant data will be made available by the corresponding author providing a methodologically sound proposal.

## Supplementary Materials

The following supporting information can be downloaded at: https://www.mdpi.com/article/10.3390/jcm13226803/s1, Tables S1–S15: Sensitivity analyses for primary analysis, sensitivity analyses for logistic regression analyses of primary endpoint, sensitivity analyses for the analysis of the secondary endpoint SSI classification, and further analysis.

## Appendix A

To obtain the imputed primary endpoint values, we estimated a logistic regression model for the primary endpoint with factor treatment and additional predictors of age, BMI, length of incision and orientation of incision. To estimate the imputation model, we used only data of those patients for whom no SSI was reported on day 3. Then, we performed a Receiver Operating Curve (ROC) analysis to find the best threshold for classifying a patient as having an SSI based on the imputation model. Finally, we obtained the model predictions for the 12 patients based on their predictor values and treatment group and obtained the imputed values according to the best classification threshold. The imputation of the secondary endpoint SSI classification was conducted in a similar way, using an ordered logistic regression model, from which we could directly predict the SSI class for the 12 patients with missing primary endpoints. To ensure consistency with the imputed primary endpoint values, we used the model prediction to impute the SSI classification only for those patients, for whom the imputed value of the primary endpoint indicated that there was an SSI within 30 days. For those patients, for whom the imputed value of the primary endpoint indicated that there was no SSI, the SSI classification was imputed accordingly as “no SSI”. Moreover, we assessed the stability of our results based on analyses of optimistic and pessimistic imputations for the missing endpoint data of all binary endpoints as well as SSI classification.
