# Supplementary material for: Do Antibacterial Skin Sutures Reduce Surgical Site Infections After Elective Open Abdominal Surgery?—A Prospective, Randomized Controlled Single-Center Trial
Source: J Clin Med. 2024 Nov 12;13(22):6803. doi: 10.3390/jcm13226803 (PMC11594667; doi:10.3390/jcm13226803)
Supplement: Supplementary file 1 [file jcm-13-06803-s001.zip › jcm-3263120-supplementary.pdf]

Supplementary Online Content

Sensitivity analyses for primary analysis 2

Table S1 Worst-case imputation model of the primary endpoint 2

Table S2 Best-case imputation model of the primary endpoint 2

Table S3 FAS patients only with certain primary endpoint 2

Sensitivity analyses for logistic regression analyses of primary endpoint 2

Table S4 Worst-case imputation model of the primary endpoint: univariable logistic regression model based on FAS data (N = 358) 2

Table S5 Worst-case imputation model of the primary endpoint: multivariable logistic regression model based on FAS data (N = 358) 3

Table S6 Best-case imputation model of the primary endpoint: univariable logistic regression model based on FAS data (N = 358) 3

Table S7 Best-case imputation model of the primary endpoint: multivariable logistic regression model based on FAS data (N = 358) 3

Table S8 FAS patients with certain primary endpoint/ PPS 3

Table S9 logistic regression model based on those FAS data with certain primary endpoint/PPS (N = 346) 4

Sensitivity analyses for the analysis of the secondary endpoint SSI classification 4

Table S10 Worst-case imputation model of SSI classification: univariable logistic regression model based on FAS data with Worst-case imputation of the SSI classification. (N = 358) 4

Table S11 Worst-case imputation model of SSI classification: multivariable logistic regression model based on FAS data with Worst-case imputation of the SSI classification. (N = 358) 4

Table S12 Best-case imputation model of SSI classification: univariable logistic regression model based on FAS data with Worst-case imputation of the SSI classification. (N = 358) 5

Table S13 Best-case imputation model of SSI classification: multivariable logistic regression model based on FAS data with Worst-case imputation of the SSI classification. (N = 358) 5

Further analysis 5

Table S14 Primary analysis based on the PPS 5

Table S15 Multivariable logistic regression model based on PPS. (N = 346) 5

Sensitivity analyses for primary analysis

Table S1 Worst-case imputation model of the primary endpoint

Standard suture SSI rate<br>Antibacterial suture SSI rate	30/180 = 16.7% [11.9%, 22.8%]<br>34/178 = 19.1% [14%, 25.5%]
Difference in SSI rates	2.4% [-5.5%, 10.4%]
P = of χ^2 test	.548

When assuming that those FAS patients with unclear primary endpoint all had experienced an SSI within 30 days, 30 out of 180 patients randomized to receive standard skin sutures experienced an SSI within 30 days. This corresponds to an SSI rate of 16.7% (95% CI [11.9%, 22.8%]). By contrast, 34 out of 178 patients randomized to receive antibacterial skin sutures are known to have experienced an SSI within 30 days. This corresponds to an SSI rate of 19.1% (95% CI [14%, 25.5%]). Thus, contrary to what had been expected, the SSI rate was higher in patients receiving antibacterial sutures than in the control group. The risk difference amounts to 2.4% (95% CI [5.5%, 10.4%]). However, this difference is not statistically significant at the 5%-level, as the null hypothesis of the χ2-test cannot be rejected (P=0.5478).

Table S2 Best-case imputation model of the primary endpoint

Standard suture SSI rate<br>Antibacterial suture SSI rate	22/180 = 12.2% [8.2%, 17.8%]<br>30/178 = 16.9% [12.1%, 23%]
Difference in SSI rates	4.6% [-2.7%, 11.9%]
P = of χ^2 test	0.214

When assuming that those FAS patients with unclear primary endpoint had not experienced an SSI within 30 days, 22 out of 180 patients randomized to receive standard skin sutures experienced an SSI within 30 days. This corresponds to an SSI rate of 12.2% (95% CI [8.2%, 17.8%]). By contrast, 30 out of 178 patients randomized to receive antibacterial skin sutures are known to have experienced an SSI within 30 days. This corresponds to an SSI rate of 16.9% (95% CI [12.1%, 23%]). Thus, contrary to what had been expected, the SSI rate was higher in patients receiving antibacterial sutures than in the control group. The risk difference amounts to 4.6% (95% CI [2.7%, 11.9%]). However, this difference is not statistically significant at the 5%-level, as the null hypothesis of the χ2-test cannot be rejected (P=0.2137).

Table S3 FAS patients only with certain primary endpoint

Standard suture SSI rate<br>Antibacterial suture SSI rate	22/172 = 12.8% [8.6%, 18.6%]<br>30/174 = 17.2% [12.4%, 23.5%]
Difference in SSI rates	4.5% [-3.1%, 12%]
P = of χ^2 test	0.247

When considering only those FAS patients, for whom it is clearly known whether or not they experienced an SSI within 30 days, 22 out of 172 patients randomized to receive standard skin sutures experienced an SSI within 30 days. This corresponds to an SSI rate of 12.8% (95% CI [8.6%, 18.6%]). By contrast, 30 out of 174 patients randomized to receive antibacterial skin sutures are known to have experienced an SSI within 30 days. This corresponds to an SSI rate of 17.2% (95% CI [12.4%, 23.5%]). Thus, contrary to what had been expected, the SSI rate was higher in patients receiving antibacterial sutures than in the control group. The risk difference amounts to 4.5% (95% CI [-3.1%, 12%]). However, this difference is not statistically significant at the 5%-level, as the null hypothesis of the χ2-test cannot be rejected (P =0.2467).

Sensitivity analyses for logistic regression analyses of primary endpoint

Table S4 Worst-case imputation model of the primary endpoint: univariable logistic regression model based on FAS data (N = 358)

	Odds ratio [Yes vs. No]	95% CI
Antibacterial suture	1.18	[0.69, 2.03]

Table S5 Worst-case imputation model of the primary endpoint: multivariable logistic regression model based on FAS data (N = 358)

	Odds ratio [Yes vs. No]	95% CI
Antibacterial suture	1.11	[0.64, 1.95]
Age	1.02	[0.99, 1.04]
BMI	0.99	[0.93, 1.05]
Length of incision	1.03	[0.98, 1.09]
Orientation of incision - longitudinal	1.63	[0.85, 3.11]
Orientation of incision - both	1.38	[0.50, 3.81]
CDC wound classification - clean contaminated	1.51	[0.69, 3.31]
CDC wound classification - contaminated/ dirty	0.00	[0.00, Inf]
Duration of operation	1.00	[1.00, 1.01]
Blood loss	1.00	[1.00, 1.00]
Colorectal procedure - yes	1.03	[0.50, 2.12]

Table S6 Best-case imputation model of the primary endpoint: univariable logistic regression model based on FAS data (N = 358)

	Odds ratio [Yes vs. No]	95% CI
Antibacterial suture	1.46	[0.80, 2.64]

Table S7 Best-case imputation model of the primary endpoint: multivariable logistic regression model based on FAS data (N = 358)

	Odds ratio [Yes vs. No]	95% CI
Antibacterial suture	1.34	[0.72, 2.48]
Age	1.03	[1.00, 1.05]
BMI	1.01	[0.95, 1.07]
Length of incision	1.03	[0.97, 1.10]
Orientation of incision - longitudinal	1.94	[0.96, 3.92]
Orientation of incision - both	1.44	[0.46, 4.50]
CDC wound classification - clean contaminated	1.84	[0.74, 4.54]
CDC wound classification - contaminated/ dirty	.00	[0.00, Inf]
Duration of operation	1.00	[1.00, 1.01]
Blood loss	1.00	[1.00, 1.00]
Colorectal procedure - yes	1.34	[0.61, 2.94]

Table S8 FAS patients with certain primary endpoint/ PPS

	Odds ratio [Yes vs. No]	95% CI
Antibacterial suture	1.42	[0.78, 2.58]

Table S9 logistic regression model based on those FAS data with certain primary endpoint/PPS (N = 346)

	Odds ratio [Yes vs. No]	95% CI
Antibacterial suture	1.31	[0.71, 2.44]
Age	1.02	[1.00, 1.05]
BMI	1.01	[0.94, 1.07]
Length of incision	1.03	[0.97, 1.10]
Orientation of incision - longitudinal	1.89	[0.94, 3.82]
Orientation of incision - both	1.42	[0.45, 4.44]
CDC wound classification - clean contaminated	1.78	[0.72, 4.39]
CDC wound classification - contaminated/ dirty	0.00	[0.00, Inf]
Duration of operation	1.00	[1.00, 1.01]
Blood loss	1.00	[1.00, 1.00]
Colorectal procedure - yes	1.29	[0.59, 2.83]

Sensitivity analyses for the analysis of the secondary endpoint SSI classification

Table S10 Worst-case imputation model of SSI classification: univariable logistic regression model based on FAS data with Worst-case imputation of the SSI classification. (N = 358)

	Odds ratio [> vs. <=]	95% CI
Antibacterial suture	1.23	[0.72, 2.10]

Table S11 Worst-case imputation model of SSI classification: multivariable logistic regression model based on FAS data with Worst-case imputation of the SSI classification. (N = 358)

	Odds ratio [> vs. <=]	95% CI
Antibacterial suture	1.22	[0.70, 2.11]
Age	1.01	[0.99, 1.04]
BMI	0.98	[0.92, 1.03]
Length of incision	1.05	[1.00, 1.10]
Orientation of incision - longitudinal	1.23	[0.68, 2.21]
Orientation of incision - both	1.10	[0.41, 2.94]

Table S12 Best-case imputation model of SSI classification: univariable logistic regression model based on FAS data with Worst-case imputation of the SSI classification. (N = 358)

	Odds ratio [> vs. <=]	95% CI
Antibacterial suture	1.49	[0.82, 2.69]

Table S13 Best-case imputation model of SSI classification: multivariable logistic regression model based on FAS data with Worst-case imputation of the SSI classification. (N = 358)

	Odds ratio [> vs. <=]	95% CI
Antibacterial suture	1.43	[0.78, 2.61]
Age	1.02	[1.00, 1.05]
BMI	1.00	[0.94, 1.06]
Length of incision	1.04	[0.99, 1.10]
Orientation of incision - longitudinal	1.43	[0.76, 2.71]
Orientation of incision - both	1.15	[0.38, 3.44]

Further analysis

Table S14 Primary analysis based on the PPS

Standard suture SSI rate<br>Antibacterial suture SSI rate	22/172 = 12.8% [8.6%, 18.6%]<br>30/174 = 17.2% [12.4%, 23.5%]
Difference in SSI rates	4.5% [-3.1%, 12%]
P = of χ^2 test	0.247

Table S15 Multivariable logistic regression model based on PPS. (N = 346)

	Odds ratio [Yes vs. No]	95% CI
Antibacterial suture	1.31	[0.71, 2.44]
Age	1.02	[1.00, 1.05]
BMI	1.01	[0.94, 1.07]
Length of incision	1.03	[0.97, 1.10]
Orientation of incision - longitudinal	1.89	[0.94, 3.82]
Orientation of incision - both	1.42	[0.45, 4.44]
CDC wound classification - clean contaminated	1.78	[0.72, 4.39]
CDC wound classification - contaminated/ dirty	0.00	[0.00, Inf]
Duration of operation	1.00	[1.00, 1.01]
Blood loss	1.00	[1.00, 1.00]

Colorectal procedure - yes	1.29	[0.59, 2.83]

Also for the secondary endpoints, *Wound dehiscence within 30 days, SSI classification and Length of hospital stay*, the results based on the PPS are very similar to those obtained from the FAS analyses. For the secondary endpoint Re-operation due to wound dehiscence within 30 days, there were only four patients all in the same study arm in the PPS. Therefore, the regression models could not be reliably estimated. A $\chi 2$ test (with continuity correction) does not find a statistically significant difference in re-operation rates due to wound dehiscence (P =0.134).
Also, regarding Death within 30 days as endpoint, there was only one patient who died in the PPS. No statistical analysis was performed here.
